# Unleashing an Underlying Serious Cardiac Arrhythmia: A Case of Coronary Vasospasm With Atypical Presentation

**DOI:** 10.7759/cureus.54971

**Published:** 2024-02-26

**Authors:** Muhammad A Baig, Ahmed Elmogy, Yasser Hegazy, Muhammad Ghallab, Mahmoud S Ahmed

**Affiliations:** 1 Internal Medicine, Icahn School of Medicine at Mount Sinai, Queens Hospital Center, New York, USA; 2 Infectious Disease, Icahn School of Medicine at Mount Sinai, Queens Hospital Center, New York, USA; 3 Internal Medicine, Icahn School of Medicine at Mount Sinai, NYC Health and Hospitals/Queens, New York, USA; 4 Cardiology, Westchester Medical Center, New York, USA

**Keywords:** prinzmetal angina, cardiac chest pain, ventricular arrhythmia, cardiac arrhythmia, coronary artery spasm

## Abstract

This case report highlights the atypical presentation of coronary artery vasospasm in a 59-year-old patient presenting with syncope due to ventricular arrhythmia. Despite initially elevated troponin levels and non-significant lesions observed during left heart catheterization, the patient experienced recurrent chest pain and dizziness, prompting further evaluation. Ultimately, coronary vasospasm was identified as the likely differential diagnosis, supported by various diagnostic modalities including electrocardiogram, Zio patch monitoring, transthoracic echocardiogram, cardiac MRI, and CT angiography. Management involved the initiation of calcium channel blocker therapy, leading to a non-eventful follow-up in the cardiology clinic.

## Introduction

Coronary artery spasm (CAS), initially described by Dr. Myron Prinzmetal in 1959 as a "variant form of angina pectoris," encompasses terms such as Prinzmetal angina, variant angina, and vasospastic angina. Diagnostic identification often relies on electrocardiograph (EKG) findings, marked by transient ST-segment elevation (STE) coinciding with recurrent rest-induced anginal episodes. This condition stems from occlusive or sub-occlusive coronary artery spasms, with endothelial dysfunction and diminished nitric oxide secretion serving as pivotal pathophysiological factors [1]. Notably, modifiable risk factors include smoking, while associations exist with male sex, magnesium deficiency, alcohol consumption, physical and mental stress, and autonomic nervous system agents [2]. Besides the classic presentation of variant angina, coronary artery vasospasm can manifest atypically, leading to acute myocardial infarction (AMI), fatal arrhythmias (e.g., ventricular tachycardia/fibrillation (VT/VF), complete atrioventricular block (AV-B)), or sudden cardiac death. Vasodilators, namely, nitrates and calcium channel blockers (CCBs), serve as effective first-line treatments for preventing vasoconstriction episodes [3].

In this case report, we present the case of a 59-year-old patient who presented with syncope due to ventricular arrhythmia, later diagnosed as a case of coronary artery vasospasm after excluding obstructive CAS.

## Case presentation

**Figure 1:**
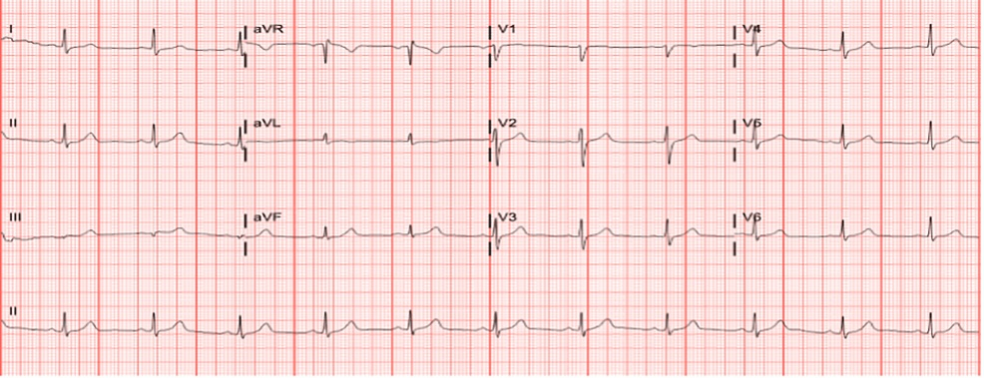
12-Lead electrocardiogram showing normal sinus rhythm on presentation to the emergency department.

We present the case of a 56-year-old gentleman, with no significant past medical history, who presented to the emergency department (ED) with acute chest pain, followed by syncope. The chest pain started a few hours before presentation with acute severe retrosternal heaviness while walking in his driveway. It lasted for a few minutes and was associated with profuse sweating. This was followed by severe dizziness and finally blacked out and was not aware of the surroundings until regaining consciousness a few minutes later to find a bystander calling emergency medical services (EMS). On presentation to ED, he was fully conscious and hemodynamically stable. EKG was done, showing no significant ST-T wave changes, and cardiac troponin was elevated (troponin T 0.09, with reference 0-0.01 ng/mL). The patient has no previous medical or surgical history. However, he had a family history of heart attack that happened to his father in his 40s, who had deceased during the same hospitalization without a clear thread of the circumstances. In addition, he has a history of sudden cardiac arrest from his maternal uncle. Three days later, left heart catheterization (LHC) was done, showing moderate to severe eccentric left main (LM) stenosis, severe proximal left anterior descending (LAD), and first diagonal (D1) disease. The lesions were judged to be hemodynamically non-significant (instantaneous wave-free ratio (iFR) 0.99 for LM and 0.91 for proximal LAD). Based on these findings, the patient was discharged on appropriate medical therapy (aspirin, high-intensity statins, angiotensin receptor blockers (ARB), and beta-blockers). He was sent home with a Zio AT patch to monitor for arrhythmia, given the severity of the presentation, and was planned for a cardiology clinic follow-up. Two days after the discharge, he had two recurrent episodes of chest pain and dizziness lasting approximately five minutes. He requested a tele-visit appointment on the same day and was instructed to return to the ED immediately for evaluation. On ED presentation, his vitals were 115/72, heart rate of 63 beats per minute, and oxygen saturation of 98% on room air. His physical examination and blood work were within normal, apart from the rise and fall of cardiac troponin (0.06, 0.1, and 0.07). His EKG showed no changes compared to the previous admission EKG (Figure 1).

**Figure 2:**
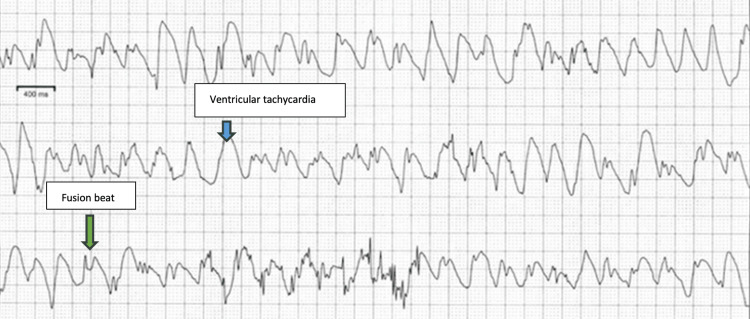
Zio patch recordings of runs of ventricular tachycardia (blue arrow) with fusion beats (green arrow).

However, a review of Zio patch rhythm strips revealed several runs of sustained and non-sustained ventricular tachycardia (VT), as shown in Figure 2.

Chest X-ray was significant for mild lung congestion. Transthoracic echocardiogram showed normal left ventricular size with normal systolic and diastolic functions (ejection fraction: 60%). The right ventricle was mildly dilated with normal systolic function. The decision was for repeat LHC, which revealed non-obstructive LM, proximal LAD, ostial D1, and proximal OM1 lesions (supported by IVUS and iFR). In addition, there was no significant gradient across the aortic valve and normal left ventricular end-diastolic pressure (LVEDP) (5 mmHg). A cardiovascular magnetic resonance imaging (CMR) was performed, which supported echo findings with the presence of late gadolinium enhancement (LGE) in the basal lateral wall and dilated left and right pulmonary arteries. However, there was no supporting evidence for mitral annular disjunction (MAD) or arrhythmogenic right ventricular cardiomyopathy (ARVC). Computed tomography (CT) angiography of the chest was done, showing no evidence of aortic dissection, aneurysm, or pulmonary embolism, and was only significant for the upper normal size of pulmonary arteries. Depending on the previous workup and exclusion of serious causes of this scenario, coronary vasospasm was the likely differential diagnosis explaining the patient’s presentation. The patient was started on a calcium channel blocker (amlodipine 5 mg once daily) and was discharged home with a non-eventful follow-up in the cardiology clinic.

## Discussion

CAS is characterized by the transient narrowing of coronary arteries, leading to myocardial ischemia. This condition presents with chest pain at rest, transient STE on the EKG, and, in severe cases, ventricular arrhythmias [3,4]. Pathogenetic mechanisms, including endothelial dysfunction, primary smooth muscle cell hyper-reactivity, abnormal growth factor production, and adventitial abnormalities, contribute to CAS [5]. It predominantly affects younger female individuals without traditional cardiovascular risk factors, besides cigarette smoking, but can also lead to severe manifestations, such as acute myocardial angina, ventricular fibrillation, or cardiac arrest [6]. Ventricular arrhythmias, notably ventricular fibrillation and ventricular tachycardia, are common during vasospastic angina crises [6]. The arrhythmic risk of coronary vasospasm ranges from 2% to 17%, varying based on baseline-risk profiles [7]. CAS can lead to life-threatening ventricular arrhythmias, as illustrated by case reports of recurrent ventricular fibrillation and polymorphic ventricular tachycardia associated with CAS [6,7]. Medical management involves calcium channel blockers as the cornerstone therapy, with long-acting nitrates or other medications added as second-line treatments if angina symptoms persist [7,8]. In patients with significant arrhythmic events due to CAS, consideration may be given to implantable cardioverter-defibrillator (ICD) placement, although its efficacy remains controversial in the context of lethal ventricular arrhythmias caused by CAS [7,9]. Clinical outcomes in patients with life-threatening ventricular arrhythmias due to CAS vary depending on medical interventions, with appropriate ICD therapy required in a significant proportion of cases [10].

## Conclusions

CAS, although typically associated with chest pain at rest and transient STE, can present with atypical symptoms such as syncope due to ventricular arrhythmia. This case underscores the importance of considering vasospastic angina as a potential diagnosis in patients with recurrent chest pain and syncope, even in the absence of significant coronary lesions. Timely recognition and appropriate management with calcium channel blockers are crucial in preventing recurrent episodes and improving clinical outcomes in patients with coronary artery vasospasm. Further research is warranted to better understand the pathophysiology of vasospastic angina and to optimize treatment strategies for this condition.
